# Characterization of reactive intermediates formation in dacomitinib metabolism and bioactivation pathways elucidation by LC-MS/MS: *in vitro* phase I metabolic investigation

**DOI:** 10.1039/c8ra06709k

**Published:** 2018-11-19

**Authors:** Mohamed W. Attwa, Adnan A. Kadi, Ali S. Abdelhameed

**Affiliations:** Department of Pharmaceutical Chemistry, College of Pharmacy, King Saud University P. O. Box 2457 Riyadh 11451 Saudi Arabia mzeidan@ksu.edu.sa +966 1146 76 220 +966 1146 70237

## Abstract

Dacomitinib (DCB) is a second generation irreversible tyrosine kinase inhibitor (TKI) that is claimed to overcome the disadvantages of the resistance developed by the first line epidermal growth factor receptor (EGFR) TKIs. In the current study, metabolites of phase I for DCB were systematically explored. DCB reactive metabolites were also investigated in rat liver microsomes in presence of potassium cyanide or methoxylamine that were employed as capturing agents for iminium reactive intermediates and aldehyde, respectively, to form stable complexes which can be detected by LC-MS/MS. As a result, four *in vitro* phase I metabolites were observed with major pathway of piperidine ring hydroxylation. Additionally, two potentially reactive intermediates, one aldehyde and one iminium ions were characterized. Two different pathways of bioactivation were ultimately proposed.

## Abstract 2

Dacomitinib (DCB) is a second generation irreversible tyrosine kinase inhibitor (TKI) that is claimed to overcome the disadvantages of the resistance developed by the first line epidermal growth factor receptor (EGFR) TKIs.

## Graphical abstract

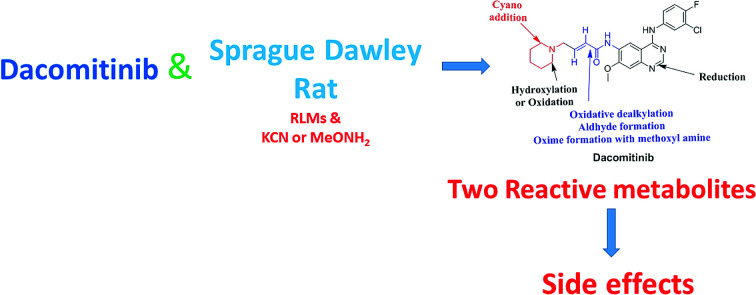

## 1. Introduction

Lung carcinoma is the principal cause of death among all cancer types. Non-small cell lung cancer (NSCLC) represents the most widespread (around 90%) of all lung cancer in patients.^1–5^ During the past few years, the epidermal growth factor receptor (EGFR) signaling pathway as a therapeutic target in NSCLC has gained more recognition.^6^ Inhibitors of the tyrosine kinase (TKIs) that affect the epidermal growth factor receptor (EGFR) are very effective against most EGFR mutations with a good therapeutic window. First line EGFR TKIs (*e.g.* erlotinib and gefitinib) had a very good response against these active mutations at the beginning.^7,8^ Unfortunately, acquired resistance (around 60%) and toxicities that occurred in treatment^9,10^ reduce their therapeutic efficacies.^11,12^

DCB is a second generation irreversible EGFR TKIs that overcomes the disadvantages of the acquired resistance first line EGFR TKIs.^13–15^ When compared with gefitinib, DCB improved progression-free survival in the management of patients with positive EGFR mutation NSCLC. DCB is regarded as a new way of treatment for this medical case.^16^ The U.S. Food and Drug Administration (FDA) accepted Pfizer's application for DCB as new drug and granted priority review for the first-line management of patients with locally advanced or metastatic NSCLC with EGFR-activating mutations. On September 27^th^ 2018 FDA approved DCB tablets (VIZIMPRO™), for the first-line treatment of individuals suffering from metastatic NSCLC with EGFR exon 19 deletion or exon 21 L858R substitution mutations.^17^ A marketing authorization application for DCB was also accepted by the EMA (European Medicines Agency) for the same indication^18^

The most common severe (grade 3) adverse reactions of DCB were acne (in 14% of patients) and diarrhea (in 8% of patients). In spite of its increased adverse reactions particularly in the skin and gastrointestinal tract, the potent activity of DCB allowed for consideration of this effective therapy in EGFR-positive NSCLC.^19–21^ Proteins in the body can be modified by covalent binding to reactive metabolites that is regarded as the primary step in organ toxicities.^22,23^ In most cases, such reactive intermediates are produced through reactions of phase I metabolism. The reactive intermediates in drug metabolism can cause many side effects. As a result of their transient nature, a trapping agent is always used to trap reactive intermediates to facilitate the formation of stable adducts. The formed adducts can be isolated from the incubation mixture, detected and characterized by LC-MS/MS.^24,25^

Chemically the structure of DCB encompasses a piperidine ring and a butenamide group. Drugs contains piperidine ring are subjected to bioactivation that forms iminium ions intermediates which may be stabilized by KCN forming cyano adducts. Butenamide group undergoes bioactivation through oxidative dealkylation developing an unstable reactive aldehyde that can be stabilized by reaction with methoxylamine forming oxime.^26–28^ These stabilized adducts may be extracted, separated and identified using LC-MS/MS.^24–26,29,30^

Upon literature review, one article was found that focused primarily on the pharmacokinetics of dacomitinib in human.^31^ Such study was aimed at characterizing the principal elimination routes of dacomitinib in humans. The study did not involve complete metabolic profiling with structural elucidation of dacomitinib metabolites. In contrast, the main target of the present work is reactive metabolites screening to provide a possible reason for toxicity of DCB *via in vitro* metabolic studies. Reactive metabolites cannot be determined *in vivo* because as soon as they are formed, they will bind to endogenous materials as DNA or proteins that cannot be detected by mass spectrometry. The basic outcome of this work is identification and characterization of two possible reactive metabolites: one aldehyde and one iminium ion with description of structure details. Bioactivation pathways were also proposed. Four *in vitro* phase I dacomitinib metabolites were also identified. The presence of such reactive intermediates in DCB metabolism may be the reason of its side effects as they are regarded the first step in drug-induced organ toxicities.^32^

## 2. Chemicals and methods

### 2.1. Chemicals

HPLC grade solvents were utilized throughout the entire study. Dacomitinib reference standard was acquired from Med Chem. Express (Princeton, NJ, USA). Acetonitrile (ACN), ammonium formate (NH_4_COOH), potassium cyanide (KCN), methoxyl amine (MeONH_2_) and formic acid (HCOOH) were procured from Sigma-Aldrich (USA). Water (ultrapure) was taken from in-house Milli-Q plus purification system (Millipore, Midford, MA USA). Rat liver microsomes (RLMs) were acquired in-house using Sprague Dawley rats.^33–36^ The used rats were attained from the experimental animal care center at college of Pharmacy, King Saud University (KSA). The University's Ethics Review Committee approved the animal experimental design.

### 2.2. Chromatography

The optimized LC-MS/MS chromatographic parameters for the separation and elucidation of incubation mixture extract components are listed in Table 1.

**Table 1:** Adjusted parameters of the proposed LC-MS/MS methodology

LC parameters			MS/MS parameters	
HPLC	Agilent 1200		Mass spectrometer	Agilent 6410 QqQ
Gradient mobile phase	A: 10 mM NH_4_COOH in H_2_O		Ionization source	Positive ESI
	B: ACN			Drying gas: N_2_ gas
	Flow rate: 0.4 mL min^−1^			Flow rate (12 L min^−1^)
	Run time: 75 min			Pressure (60 psi)
Agilent eclipse plus C_18_ column	Length	150 mm		ESI temperature: 350 °C
	Internal diameter	2.1 mm		Capillary voltage: 4000 V
	Particle size	3.5 µm	Collision gas	High purity N_2_
	Temperature:	22 ± 1 °C	Modes	Mass scan and product ion (PI)
Gradient system	Time	% B	Analyte	DCB and its reactive metabolites
	0	5		
	5	5	Mass parameters	Fragmentor voltage: 140 V
	40	60		
	70	90		
	75	5		Collision energy of 20 eV

### 2.3. RLMs incubations

Metabolic reactions were done by incubation 30 µM of DCB with 1.0 mg mL^−1^ RLMs in addition to 50 mM Na/K phosphate buffer (pH 7.4) that contains 3.3 mM MgCl_2_. Incubation was done at 37 °C for 2 h in a temperature-controlled rocking water bath. The addition of 1.0 mM NADPH started the metabolic reactions while the addition of 2 mL ice-cold ACN was utilized to quench those reaction.

### 2.4. Extraction and purification method of DCB RLMs incubations

Protein precipitation method by ACN was performed to extract and purify DCB, its *in vitro* phase I metabolites and its adduct from RLMs incubation mixtures. Removal of proteins was done by centrifugation of metabolic mixtures at 9000*g* for 15 min at 4 °C. Supernatants were transferred to clean tubes. Evaporation of the supernatants was done under nitrogen stream. The mobile phase was used to reconstitute the residues and 1 mL from each was put in HPLC vial. Fifteen µL were injected into LC system.^36–38^

### 2.5. Characterization of DCB bioactive intermediates

The same metabolic incubation of DCB with RLMs was repeated in the presence of 2.5 mM methoxyl amine and 1.0 mM KCN to seize the aldehyde intermediates and reactive iminium intermediates, respectively. Each experiment was repeated three times to verify the outcomes. Three controls were prepared to confirm the results. Control 1 (C1) contains all incubation ingredients including DCB and RLMs except NADH and nucleophiles to confirm *in vitro* phase I metabolites. C2 contains all incubation ingredients including RLMs except NADH and DCB to confirm the absence of any interference from indigenous materials. C3 contains all incubation ingredients including NADH and DCB and nucleophiles (KCN or MeONH_2_) except RLMs to further proof that the reactive metabolites formation occurred only through metabolic reaction.

### 2.6. Identification of DCB reactive metabolites

Full mass scan and extracted ion chromatograms were utilized to annotate metabolites in the incubation mixtures, while product ion (PI) was employed to identify DCB *in vitro* metabolites, adducts and reactive intermediates formed in DCB metabolism. Locating metabolites in metabolic mixture extract chromatogram was performed by EIC of *m*/*z* of the proposed DCB metabolites.

## 3. Results and discussion

### 3.1. PI study of DCB

**Fig. 1:**
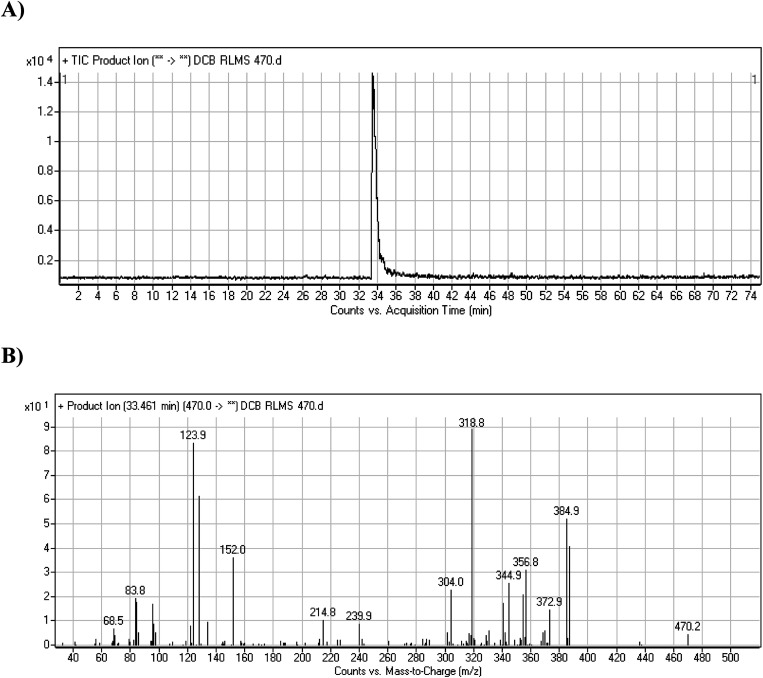
PI chromatogram of ion at *m*/*z* 470 showing DCB peak at 33.5 min (A), PIs of DCB (B).

**Scheme 1:**
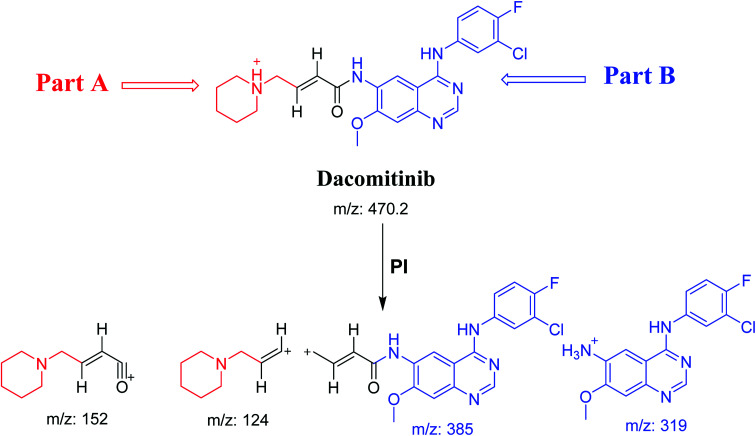
PIs of DCB.

DCB chromatographic peak appears at 33.5 min in PI chromatogram (Fig. 1A). Collision induced dissociation (CID) of molecular ion at *m*/*z* 470 generates four characteristic fragment ions at *m*/*z* 385, *m*/*z* 319, *m*/*z* 152 and *m*/*z* 124 (Fig. 1B). Fragment ions at *m*/*z* 152 and *m*/*z* 124 were used as indicator for any metabolic change in part A of DCB chemical structure while fragment ions at *m*/*z* 385 and *m*/*z* 319 were used as indicator for any metabolic change in part B of DCB chemical structure (Scheme 1).

### 3.2. Identification of *in vitro* DCB metabolites

After extraction and purification of RLMs incubations, fifteen µL were injected into LC-MS/MS instrument. Major metabolic phase I pathway for DCB was piperidine ring hydroxylation. One methoxyl amine conjugate and one cyano adduct were characterized after incubation with RLMs with 2.5 mM methoxylamine and 1.0 mM KCN, respectively (Table 2).

**Table 2:** *In vitro* phase I and reactive metabolites of DCB

	MS scan	Most abundant fragment ions	Rt. (min)	Metabolic reaction
DCB	470	385, 319, 152, 124	33.5	Parent drug
DCB484	484	385, 319, 166, 144	39.5	α Oxidation at piperidine ring
DCB472	472	387, 321, 152, 124	32.2	Reduction at quinazoline ring
DCB486a	486	387, 166, 138	39.2	α Oxidation at piperidine ring and reduction at quinazoline ring
DCB486b	486	385, 319, 168	30.4	Hydroxylation at piperidine ring
DCB495	495	385, 319, 122	52.5	Cyano adduct
DCB362	362	332, 305	38.7	Methoxyl amine conjugate (oxime)

#### 3.2.1. DCB484 phase I metabolite

**Fig. 2:**
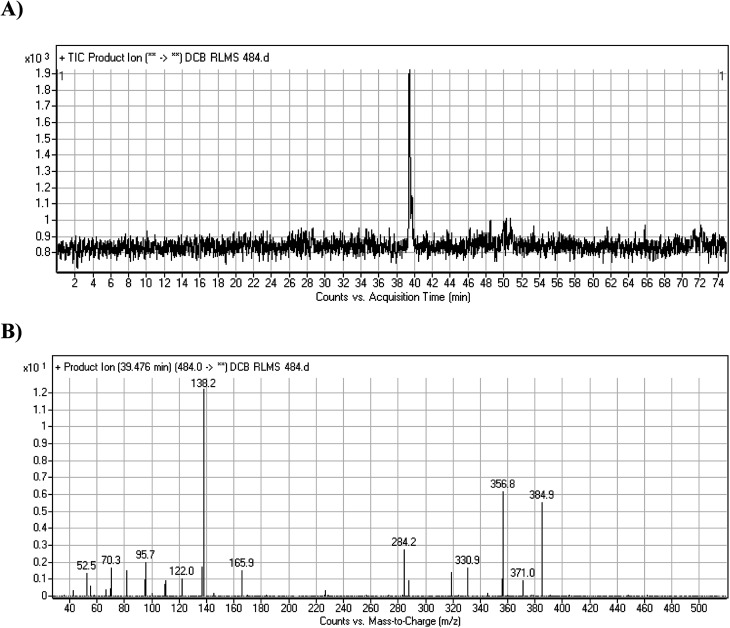
PI chromatogram of ion at *m*/*z* 484 showing DCB484 peak at 39.5 min (A), PIs of DCB484 (B).

**Scheme 2:**
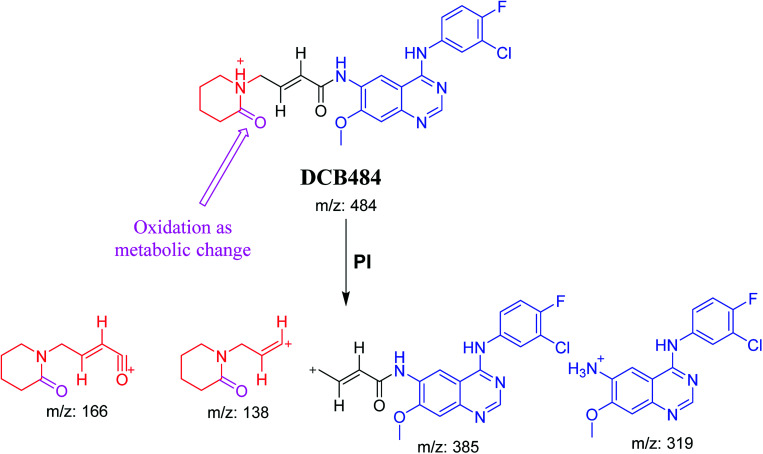
PIs of DCB484.

DCB484 chromatographic peak appears at 39.5 min in PI chromatogram (Fig. 2A). CID of molecular ion at *m*/*z* 484 generates four characteristic fragment ions at *m*/*z* 385, *m*/*z* 319, *m*/*z* 166 and *m*/*z* 138 (Fig. 2B). In contrast to DCB fragmentation, fragment ions at *m*/*z* 166 and *m*/*z* 138 indicated that metabolic alteration took place in part A of DCB chemical structure while fragment ions at *m*/*z* 385 and *m*/*z* 319 indicated that no metabolic change occurred in part B of DCB chemical structure. α-Oxidation metabolic reaction was hypothesized to take place in piperidine ring (Scheme 2).

#### 3.2.2. DCB472 phase I metabolite

**Fig. 3:**
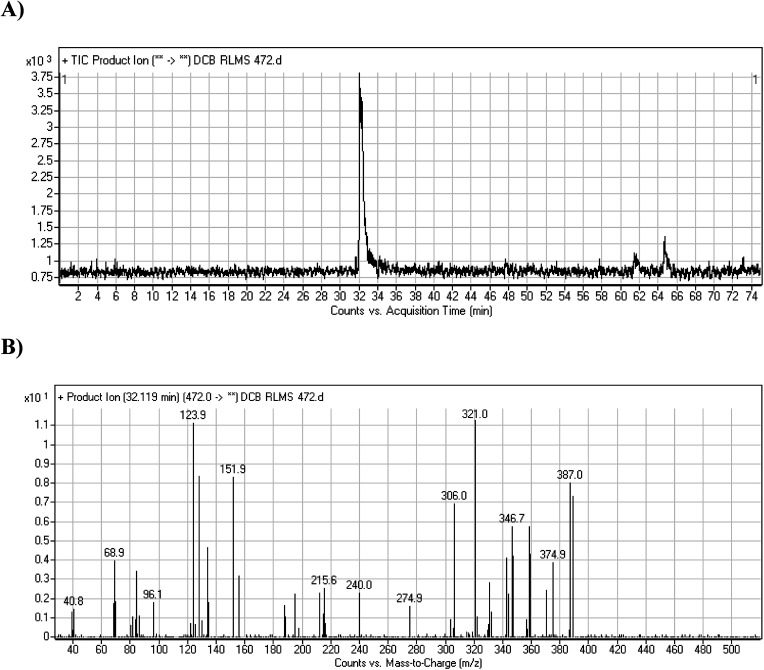
PI chromatogram of ion at *m*/*z* 472 showing DCB472 peak at 32.1 min (A), PIs of DCB472 (B).

**Scheme 3:**
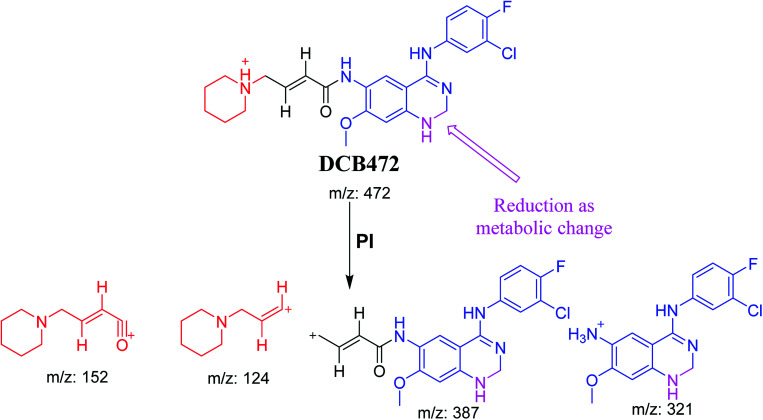
PIs of DCB472.

DCB472 chromatographic peak appears at 32.1 min in PI chromatogram (Fig. 3A). CID of molecular ion at *m*/*z* 472 generates four characteristic fragment ions at *m*/*z* 387, *m*/*z* 321, *m*/*z* 152 and *m*/*z* 124 (Fig. 3B). In contrast to DCB fragmentation, fragment ions at *m*/*z* 152 and *m*/*z* 124 indicated that no metabolic modification took place in part A of DCB chemical structure while fragment ions at *m*/*z* 387 and *m*/*z* 321 indicated that there was a metabolic change in part B of the DCB chemical structure. Reduction metabolic reaction was suggested to take place in quinazoline ring (Scheme 3).

#### 3.2.3. DCB486 phase I metabolites (a to b)

**Fig. 4:**
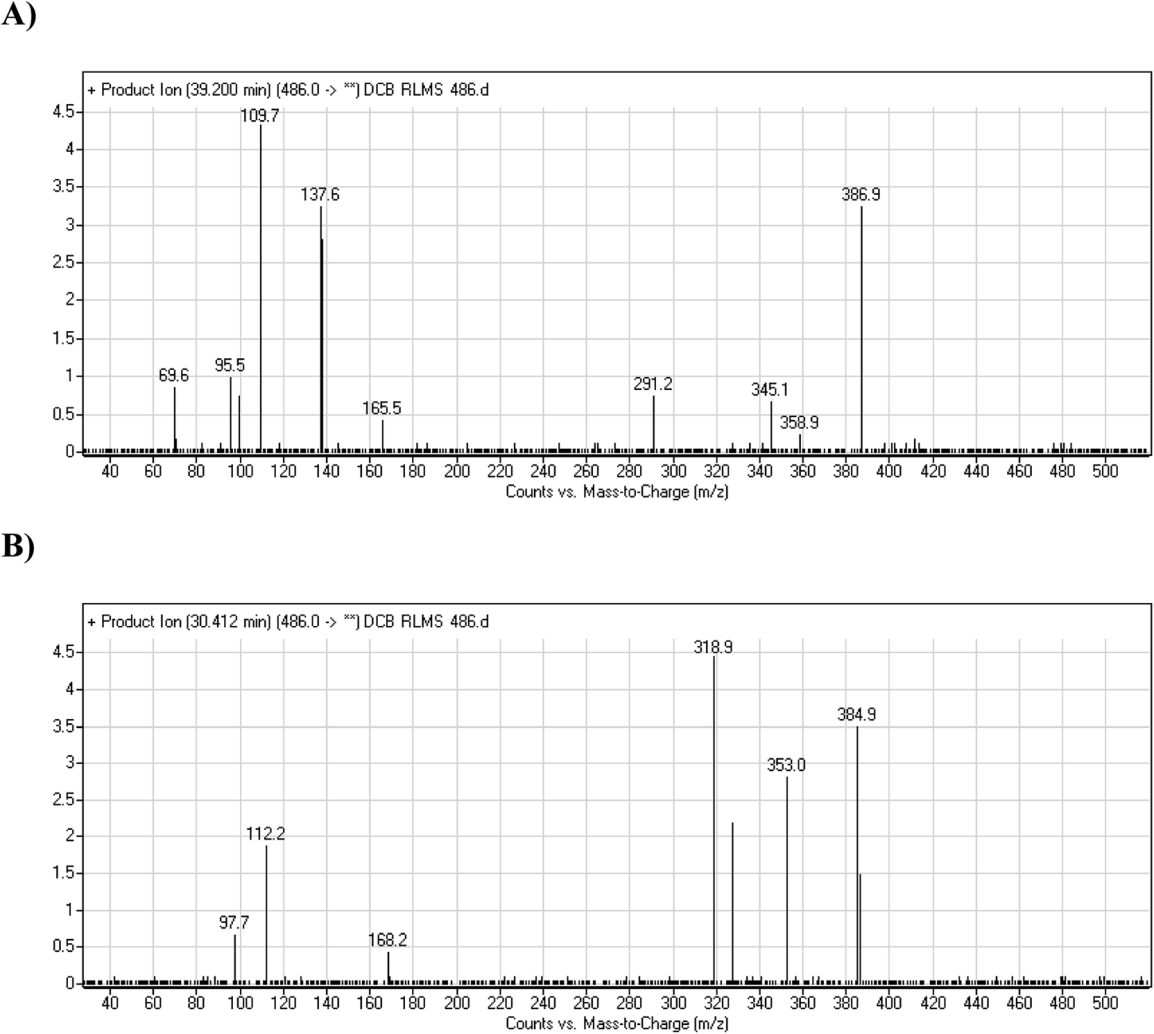
PIs of DCB486a (A), PIs of DCB486b (B).

**Scheme 4:**
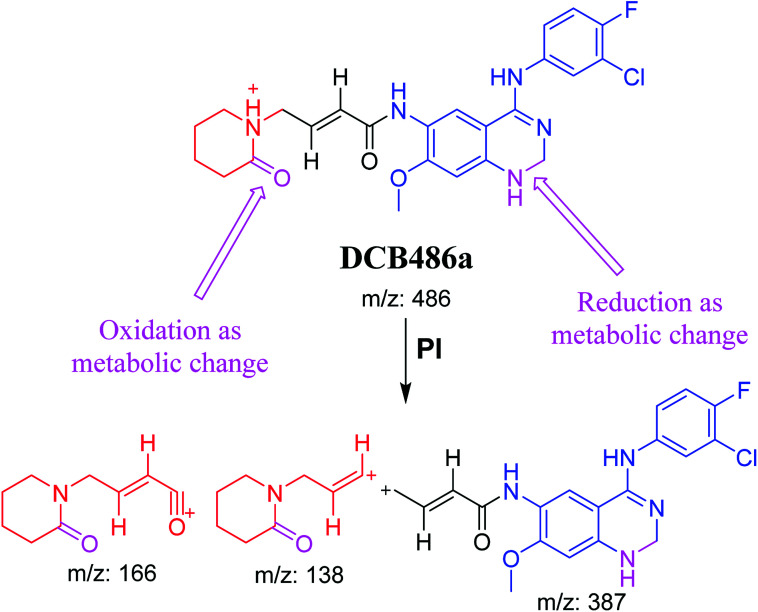
PIs of DCB486a.

DCB486 chromatographic peaks appear at 39.2 min and 30.4 min in the PI chromatogram. For DCB486a, CID of molecular ion at *m*/*z* 486 generated three characteristic ions at *m*/*z* 387, *m*/*z* 166 and *m*/*z* 138 (Fig. 4A). In contrast to DCB fragmentation, fragment ions at *m*/*z* 166 and *m*/*z* 138 indicated that α oxidation metabolic alteration took place in part A of DCB chemical structure while fragment ions at *m*/*z* 387 indicated that another reduction metabolic change occurred in part B of DCB chemical structure. α-Oxidation metabolic reaction was assumed to occur in piperidine ring in addition to a reduction reaction in quinazoline ring (Scheme 4).

**Scheme 5:**
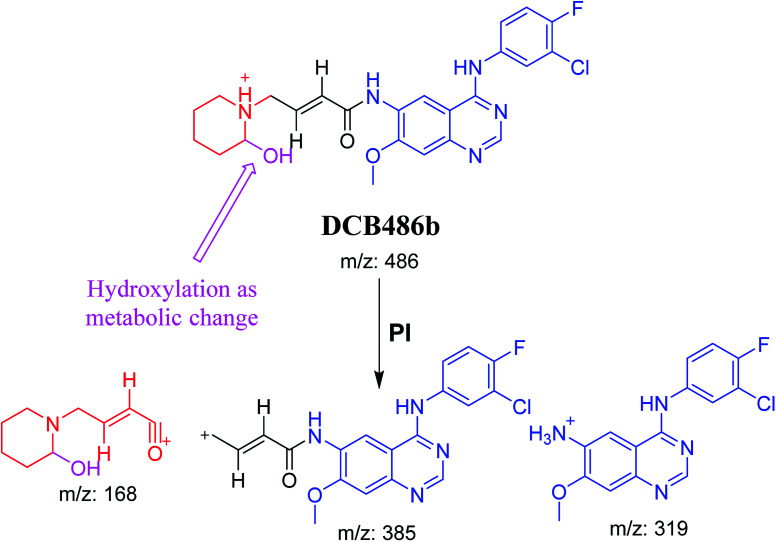
PIs of DCB486b.

In case of DCB486b, (CID) of molecular ion at *m*/*z* 486 generates three characteristic fragment ions at *m*/*z* 385, *m*/*z* 319 and *m*/*z* 168 (Fig. 4B). In comparison to DCB fragmentation, fragment ions at *m*/*z* 385 and *m*/*z* 319 indicated that α oxidation metabolic change occurred in part B of DCB chemical structure while fragment ions at *m*/*z* 168 indicated that another reduction metabolic change occurred in part A of DCB chemical structure. Hydroxylation reaction was hypothesized to metabolically occur in piperidine ring of DCB (Scheme 5).

#### 3.2.4. DCB495 cyano adduct

**Fig. 5:**
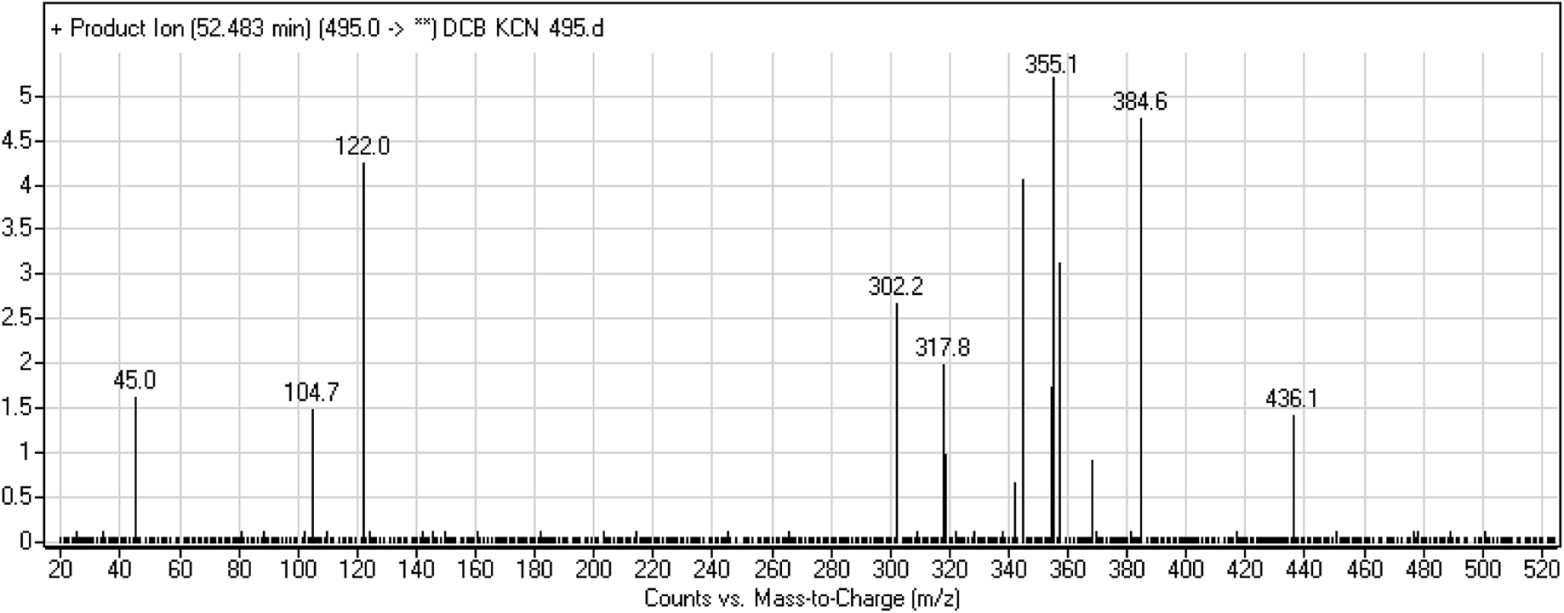
PI mass spectrum of DCB495.

**Scheme 6:**
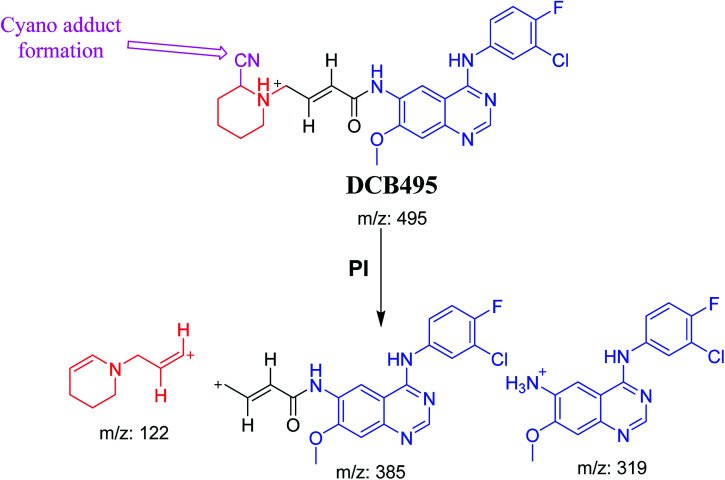
PIs of DCB495.

DCB495 chromatographic peak appears at 52.5 min in PI chromatogram. CID of molecular ion at *m*/*z* 495 generates three characteristic fragment ions at *m*/*z* 385, *m*/*z* 319 and *m*/*z* 122 (Fig. 5). In contrast to DCB fragmentation, fragment ions at *m*/*z* 385 and *m*/*z* 319 indicated that no metabolic effect on part A of the DCB chemical structure while fragment ions at *m*/*z* 122 indicated that a metabolic modification took place in part B of DCB chemical structure that signified a loss of HCN. In bioactivation of similar piperidine containing drugs,^39,40^ piperidine ring carbons was hypothesized to undergo bioactivation followed by attack by cyanide ion as explained in Scheme 6.

#### 3.2.5. DCB362 oxime adduct of avitinib

**Fig. 6:**
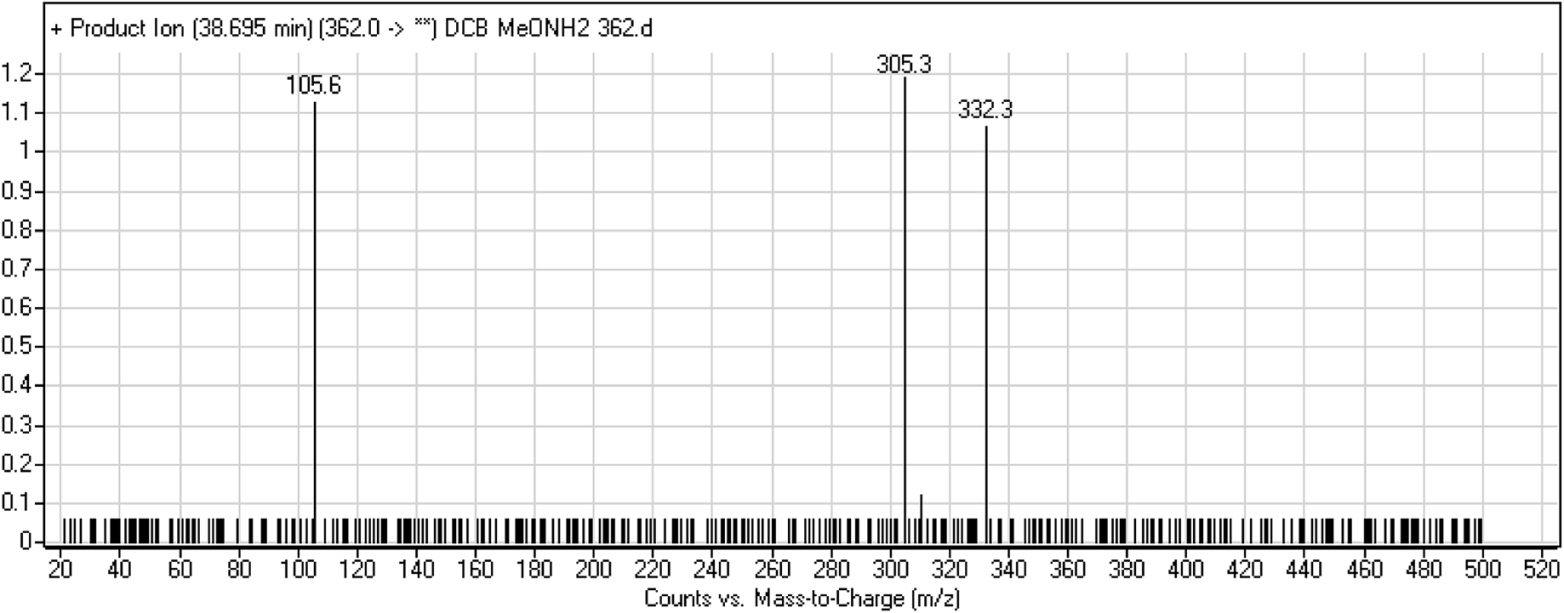
PI mass spectrum of DCB362.

**Scheme 7:**
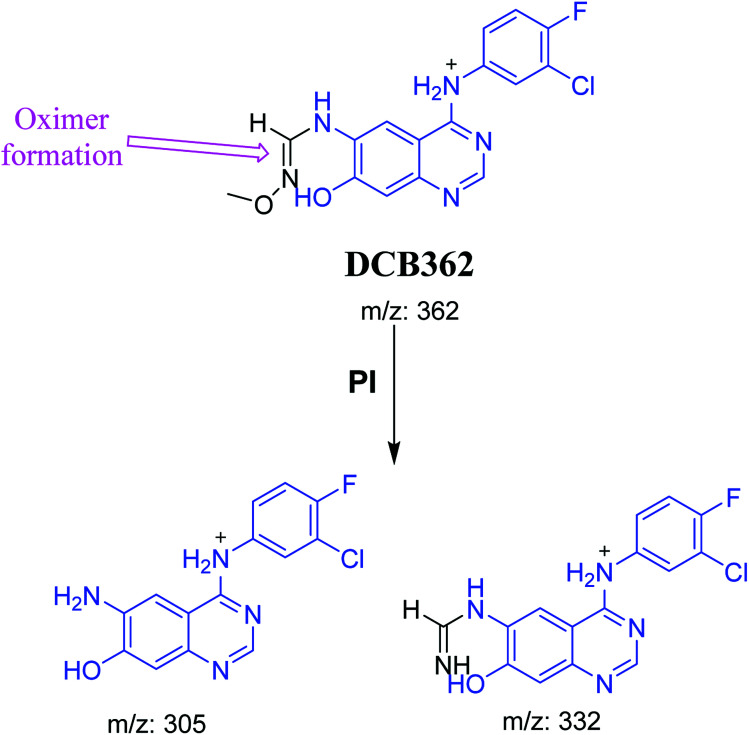
PIs of DCB362.

DCB362 chromatographic peak appears at 38.69 min in PI chromatogram. CID of the molecular ion at *m*/*z* 362 generates three characteristic fragment ions at *m*/*z* 332, *m*/*z* 305 and *m*/*z* 106 (Fig. 6). In comparison to DCB fragmentation, fragment ions at *m*/*z* 332 and *m*/*z* 305 indicated that no metabolic change occurred in part B of DCB chemical structure contains butenamide group undergoes bioactivation through oxidative dealkylation to form unstable reactive aldehyde that can be stabilized by reaction with methoxylamine forming oxime. The formation of DCB362 oxime indicated that aldehyde intermediates were formed in the metabolism of DCB (Scheme 7).

### 3.3. Proposed pathways of bioactivation of DCB

**Scheme 8:**
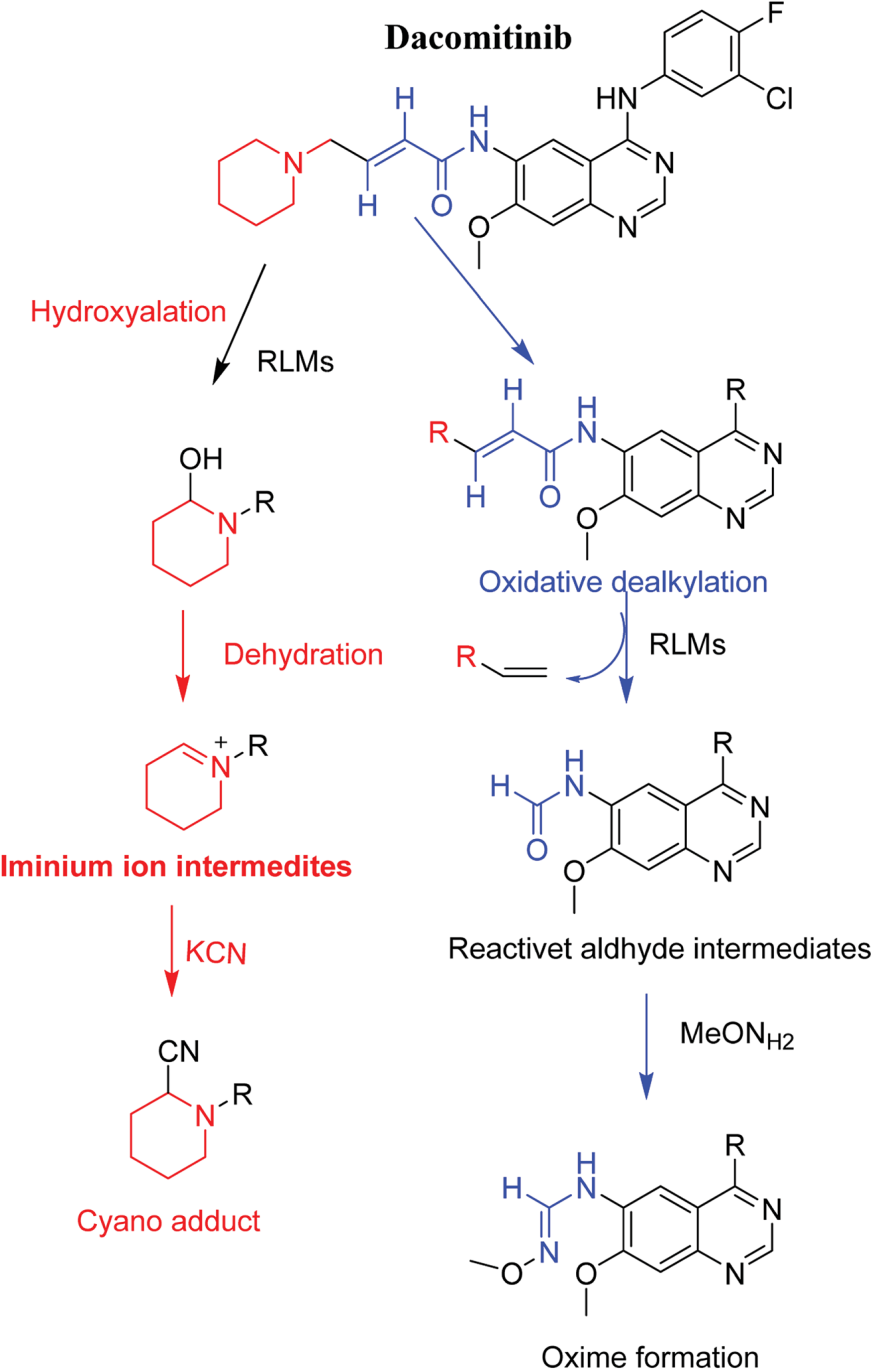
Proposed pathways for DCB bioactivation.

Scheme 8 showed the different pathways for bioactivation of DCB. DCB495 cyanide adduct confirmed iminium intermediate formation in piperidine ring metabolism. Hydroxylation of piperidine ring in DCB followed by dehydration led to generation of unstable and reactive iminium ions intermediates that may be captured by cyanide to form stable adduct which can be detected in LC-MS/MS. The formation pathway of iminium intermediate and bioactivation mechanism of DCB was described earlier with similar drugs encompassing cyclic tertiary amine.^36,37^ The aldehyde (DCB362) was formed by oxidative dealkylation of butenamide group and captured by methoxylamine to form a stable oxime containing compound (Scheme 8).

## 4. Conclusions

**Fig. 7:**
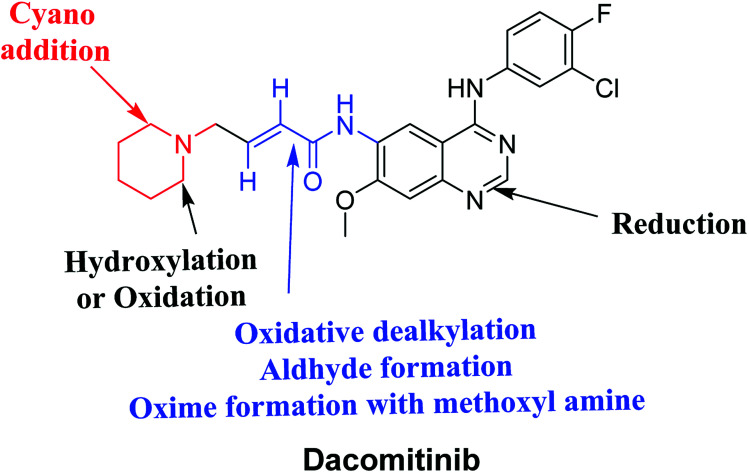
Chemical structure of DCB showing bioactivation pathways including iminium and aldehyde formation.

Four DCB metabolites were isolated and detected by LC-MS/MS *in vitro* resulting from phase I metabolic reactions. Two possible reactive metabolites: one aldehyde and one iminium ion were detected and the bio-activation pathways were proposed (Fig. 7). These reactive intermediates in DCB metabolism may be the reason of its side effects as they are considered the primary step in drug-induced organ toxicities. The results obtained in the present study paves the way for the development of new drugs with more safety profile.

## Conflicts of interest

The authors declare no conflict of interest.

## Supplementary Material
